# Telling our history: a narrative study with Indigenous elders, wisdom for the coming generation

**DOI:** 10.3389/fpubh.2026.1817753

**Published:** 2026-06-17

**Authors:** Allyson Kelley, Dolores BigFoot, Clayton Small, Phil Steven, William Snell, Gail Raines, Ruth Cedar Face

**Affiliations:** 1 Allyson Kelley & Associates PLLC, Sisters, OR, United States; 2 Native PRIDE, Fleming Island, FL, United States; 3 Doya Natsu Healing Center, Fort Washakie, WY, United States; 4 Rocky Mountain Tribal Leaders Council, Billings, MT, United States; 5 Consultant, Pryor, MT, United States; 6 Little Wound School, Kyle, SD, United States

**Keywords:** decolonizing research methodologies, generational healing, indigenous elders, qualitative method, storytelling

## Abstract

**Introduction:**

The history of AIAN people in the US has been told many times, but Western academic researchers have failed to shift that history into wisdom and uncover teachings for living in the present day. The aim of this paper is to document Indigenous elders’ childhood experiences and stories, and to record the wisdom they wish to pass on to the next generation in an accessible, written form.

**Methods:**

This study used elder-centered research methods situated within a postcolonial paradigm. Methods centered on the lived experience and values of Indigenous people and a three-step process of identifying data, developing a methodology, and validating wisdom messages in a visual model. Participants were recruited for this study through existing connections and prior research experience. Inclusion criteria for participants were: (1) elder status in their community/Tribe, (2) willingness to share a story from childhood and wisdom, and (3) validate stories and the visual model presented in this manuscript. There were two open-ended questions asked during the information gathering interviews: (1) “Tell me a story from your childhood,” and (2) “What is some wisdom you want to pass on?”

**Results:**

Fourteen stories (vignettes) are presented in this section along with a visual model that highlights primary messages (themes). Past, present, and future messages from stories are illustrated using an Indigenous Life Course timeline against the backdrop of colonial events and American Indian policy.

**Conclusion:**

This study supported the transmission and continuation of Indigenous knowledge systems, lifeways, practices, traditions, values, and experiences that are often lost or never recorded. This study illuminates the life stories of elders who possess deep resilience and strength and have experienced the devastating impacts of colonialism and the loss of land, language, values, and family systems. Combined, these wisdom messages have the potential to heal individuals, inform community-based programs, and elevate Indigenous knowledge in public health. Elders’ deep wisdom and legacy are reflected in what they wanted to share with readers, a call to action about how to live: Keep Praying. You Have A Voice. Go Back to the Old Ways, Share Your Stories About How to Live, Walk in Beauty. Love Your Family, Limit Technology, Protect the Land, and Medicines. Deal with Pain, Practice Ceremony and Self-care, Live in Balance. Pray, Help People, Commit to Something. Practice Ceremony, Forgive, Heal from Losses, Be Generous, Help Others, Seek Happiness.

"We need to tell our history. We have the instructions. We know what we're supposed to be doing."

—Indigenous Elder, Anonymous, December 2025

## Introduction

American Indian and Alaska Native (AIAN) people have existed and thrived for more than 30,000 years. They include 575 federally recognized Tribes in the United States and comprise more than 9.7 million people (2.9% of the US population) (1). AIAN Tribes have distinctive languages, knowledge systems, histories, values, land, and teachings. However, AIAN people have the lowest life expectancy in the US when compared with non-Hispanic whites, their way of life being impacted by premature and preventable deaths (2). Factors contributing to lower life expectancy include lingering impacts of colonization, trauma, substance use, poor health care, limited education, discrimination, and persistent oppression. Combined, the premature loss of AIAN people, especially elders, has interrupted the transmission of wisdom to the present and coming generations.

### History

To understand the present, we must remember the past. The history of AIAN people in the US has been recounted many times, yet Western academic researchers have fallen short of transforming that history into lived wisdom or drawing out teachings relevant to contemporary life. The existence of AIAN people can be traced back to 100,000 BC, and despite war, relocation, cultural genocide, and assimilation, they endure (Table 1). AIAN resilience, sustained through the transmission of elder wisdom, has been key to endurance.

**Table 1:** Documentation of AIAN historical events related to elder stories.

100,000 BC–AD 1491 era of First Nations
1492–1777 Colonizers and resistance
1785—Treaty of Hopewell places Native Cherokees in Georgia on land with borders
1178–1829 Reshaping America
1819—Assimilation Era Begins, Congress passed the Civilization Fund Act, the beginnings of boarding schools, cultural genocide, families destroyed, language and ceremonies lost
1830–1920 Defining Rights and Responsibilities
1830—Indian Removal Act; the government relocates Indians
1851—Indian Appropriations Act creates reservation system, moves Tribes to reservations (37)
1860–1978 Boarding School Era- Kill the Indian, Save the Man
1887—Dawes Act Divides Reservations into small plots of land for Indians
1921–1967 Citizenship, Services, and Sovereignty
1934—Indian Reorganization Act aims to restore culture, return land, and encourage self-governance
1968–2010 Renewing Native Ways (38)
1968—American Indian Movement (AIM) begins
1973—AIM takes over Wounded Knee, SD, to protest government corruption, 71 days, two killed, 12 wounded, 1,200 arrested (39)
2011—Present resistance, reparations, and reclaiming

This study occurs in the present (2025–2026) and centers on elders who have lived through key periods of this nation’s colonial history. The childhood stories they share take place during pivotal historical moments. Elders recount experiences from their own reservation life in the 1950s and 1960s, the American Indian Movement (AIM) in 1972, as well as the long-term impacts of colonial history on their loss of language and culture, the disruption of kinship systems, poverty, and ongoing discrimination.

### Indigenous story as medicine, wisdom, and praxis

Indigenous people have relied on oral storytelling traditions for thousands of years. Oral stories and traditions connect AIAN people to their language, culture, kinship systems, teachings, histories, and Creation Stories (3). Ethnographers use graphic narrative stories to address difficult or uncomfortable data and fieldwork experiences while translating research into practice and understanding (4). For example, researchers have used storytelling as evidence to document events of historical trauma among boarding school survivors (5). Nursing science has used stories to generate understanding and explanations of Indigenous health conditions, including experiences with diabetes and the holistic nature of life (6).

Stories are universal—they transcend time, space, culture, and language. A Hopi proverb says that those who tell the stories rule the world. No culture has endured without passing stories from one generation to the next. Yet a central challenge is that wisdom stories are not always preserved in written, published, or Western academic literature. Some Tribal colleges and community colleges offer creative arts and writing programs to preserve language and stories; others have elder wisdom libraries with oral histories, interviews, and cultural preservation initiatives (7). There is no single way to access elder wisdom stories and teachings. Increasingly, contemporary novels and children’s books feature ancestral teachings, language, and trickster stories. In addition, booklets, films, and modern media such as Photovoice, photo method, podcasts, and websites are used to record and share stories and engage in dialogue with listeners and readers (8). As sovereign nations, Tribes determine the processes and methods for collecting, sharing, and telling stories. However, funding and support for Tribal language and story are often limited. The following stories serve as a record of Indigenous elder life experiences. This paper aims to document Indigenous elders’ childhood stories and preserve the wisdom they wish to pass on to future generations in an accessible form. These areas were selected because much of Indigenous history and lived experience has been underdocumented or misrepresented. By centering first-person narratives, this publication honors elders’ experiences, truth, wisdom, and the prayers in their hearts. These stories give voice to lived experiences and histories, while offering guidance that can support healing for present and future generations. Readers will gain an understanding of history, colonization, and the resilience of AIAN people. The wisdom shared offers insight into how to live in the present. Ultimately, these stories and teachings form elders’ legacies; contributions intended to strengthen the health and wellbeing of generations to come.

### Research design and methods

This study used a blend of decolonized Indigenous research methods that are culturally grounded, elder-centered, and situated within a postcolonial paradigm grounded in the lived experience and values of Indigenous people (9). Following methodological guidelines for elder-entered research, this study focused on identifying informative data, developing an elder-specific methodology, and testing and validating the results (10). Methods honor the intellectual sovereignty and histories of Indigenous people (11), while reminding us that stories contain teachings that are neither quantifiable nor generalizable (12, 13).

This study and its methods build on previous research with Indigenous elders and communities, and on ways of knowing. The blending and borrowing of these approaches add depth and cultural relevance to the process and influence the messages identified regarding wisdom and intergenerational connections.

Our previous research experiences with multiple frameworks informed our approach to this research and our implicit and explicit understanding of Western-coined frameworks in the context of Indigenous spaces. Consistent with a decolonized, Indigenous-elder-centered methodology, we honor and respect these teachings and publications by briefly summarizing them here. “TribalCrit” (a Tribal framework informed by Critical Race Theory (CRT)) was used to reflect on stories as theories and as legitimate data sources that guide Indigenous ways of being (9). Tribal Participatory Research (TPR) (14) helped us explore elders’ stories through the social construction of knowledge (15–17). It also recognizes the tenets of Community-Based Participatory Research (CBPR) (18), including trust, mutual benefit, and long-term commitment, and the rebalancing of power relations. Narrative inquiry (a Western research method) (19–21) was also considered as we began this study, given previous research articles that validate and demonstrate the importance of using storytelling as a methodological framework.

### Elders/participants

The first author recruited participants for this study through existing connections and prior research experience. Inclusion criteria for participants were: (1) attained elder status in their community/Tribe, (2) willingness to share a story from childhood and wisdom, and (3) willingness to assist with validating stories and the visual model presented in this manuscript. Elders’ selection was purposeful; participants were emailed an invitation from the first author outlining the study’s parameters and the need for elders to complete the interviews. The initial goal was to interview 10 elders–seven agreed to be interviewed. Elders represented a variety of backgrounds and histories. Tribal affiliations included Navajo, Caddo, Crow, Northern Cheyenne, Lakota, and Assiniboine. One elder requested that their affiliation and wisdom be anonymous. Elders were working professionals, including a natural resource manager, an executive director, a clinical psychologist, a traditional healer, and a licensed clinical counselor. Consistent with the Indigenous values of respect, honesty, love, bravery, humility, and truth (22, 23), elders received an honorarium for their time and a small gift.

### Procedure and data

Following the CARE Principles for Indigenous Data Governance (Collective Benefit, Authority to Control, Responsibility, Ethics), and Indigenous story-telling guidelines (data) (24), the ethical approval process was guided by the CARE principles and Indigenous data sovereignty, which affirms Indigenous peoples rights to govern their own data and make decisions about the creation of data, analysis, interpretation, sharing, and storage. Approval was provided by elders who represented and willingly shared their own stories and knowledge for this publication. Here, the relationship between the elder and the first author was paramount to any external colonial review process. However, because these stories are published in a Western-dominated peer review journal that requires documented consent, elders provided written and verbal consent before interviews began.

While in-person storytelling is preferred in Indigenous spaces, the geographical distance between the elders and the first author made this unfeasible. Stories were therefore gathered via Zoom, with interviews lasting between 20 and 60 min. Elders were given the option to remain anonymous or use their real names. They were also invited to be co-authors of this manuscript–consistent with Tribal research recommendations (23–25). Elders are recognized as the authors of their own stories. Names of places, people, events, and experiences were retained in the stories, except for when elders felt the information should be masked or changed to prevent potential harm.

There were two open-ended questions asked during the interview: (1) “Tell me a story from your childhood,” and (2) “What is some wisdom you want to pass on?” The first author listened to all interviews twice and had two students aid in interview transcription. The goal of including the coming generation in the transcription was to introduce them to this wisdom and method, which are not readily available in colonized educational systems and environments (26). Files were uploaded and saved to a password-protected shared file managed by the first author.

Following elder-centered research methodologies, stories were not analyzed using traditional Western qualitative research methods, which often quantify, deduce, interpret, and generalize them into themes, codes, and frequencies (27). Reviews, listening, and reflection of elder childhood and wisdom stories were limited, inductive, reflexive, and iterative. Accordingly, the elder-centered research approach calls for identifying informative data, developing the methodology, and testing and validating (10, 28), as shown in Table 2.

**Table 2:** Elder-centered research methodology.

Elder-centered recommended methodological steps	Present study actions
Identifying informative data	Interviews with elders, reflection, reviews
Developing methodology	Reviews, remembering and applying methodology to the present study
Testing and validation	Reviews of wisdom, primary messages, and legacies presented in a visual model, reviewed, verified, and validated by elders

**Figure 1:**
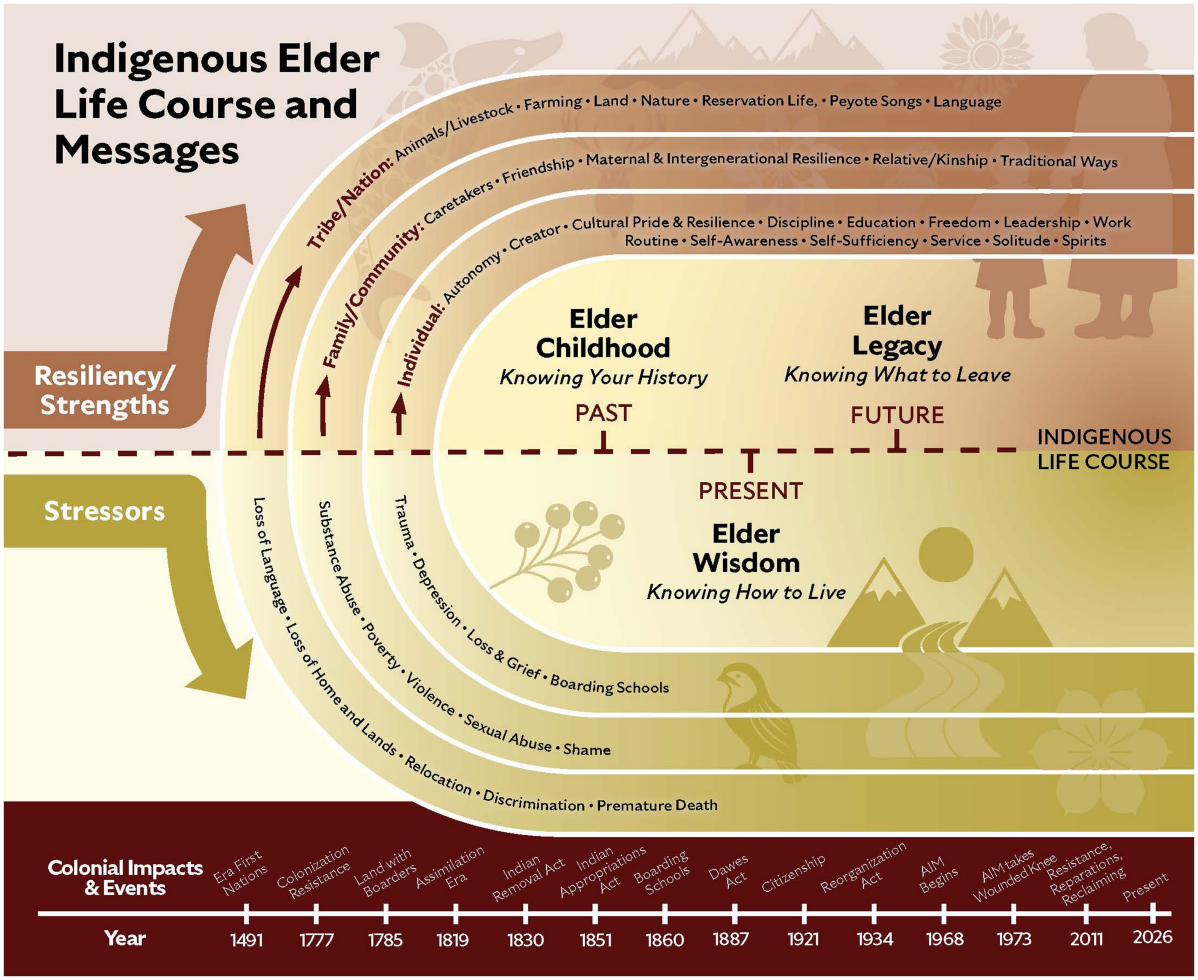
Validated visual model with messages and timeline.

Initial meanings and messages from the stories were identified by the first author through rereading all transcripts, listening to the stories multiple times, and writing key messages on paper. The first author considered the data and the Indigenous life-course perspective (29), which considers past, present, and future generations (Step 1). The visual model depicts the informative data and message, and while placing it within a socio-ecological model: Individual/self, family/community, and Tribe/nation (27). This involved identifying common meanings and messages across stories and presenting them in a visual model that is easily accessible to people from all walks of life, knowledge systems, education levels, and time. A timeline is presented in the visual model (see Figure 1) to show the impacts of colonization, treaties, and American Indian policy on elder stories, wisdom, and childhood experiences (Step 2). Consistent with Indigenous values and methods (23, 30) and Indigenous research sovereignty (31), elders were given copies of their recordings and stories, the visual model, and all information related to this research.

Selected stories and the visual model were verified and approved by the elders and the first author of this study. Here, elders own and maintain the stories (data); they determine how the data is transmitted to their families and future generations. Stories are presented as vignettes (24, 25) to understand the past, their teachings, culture, and experiences.

## Results

Fourteen stories (vignettes) are presented in this section, along with a visual model that highlights key messages validated by elders (referred to as “themes” in Western research). Past, present, and future teachings are illustrated using an Indigenous Life Course timeline (29), set against the backdrop of colonial events and American Indian policy, which have profoundly shaped US history and the lived experiences of elders today. The visual model draws on previous public health efforts that use the socio-ecological model (individual, family/community, tribal/nation) and the social determinants of health (32). Elder childhood stories reflect both resilience and strength, as well as significant adversity and stress. In this context, resilience is born from the adversity and challenges that elders have faced. At the individual level, common messages include autonomy, cultural pride, resilience, freedom, self-awareness, self-sufficiency, spirituality, and service. At the family/community level, sources of resilience included caretakers, friendship, maternal and intergenerational relationships, relatives and kinship, and traditional ways. At the Tribal/Nation level, connection to animals, gardening, land, nature, reservation life, and practices such as peyote songs and language sustained elders as they grew into healthy, balanced adults. Elders also described stressors across these levels. At the individual level, these included trauma, depression, loss, and grief, and the impacts of boarding schools. Within family and community contexts, substance abuse, violence, poverty, and sexual abuse emerged. At the Tribal/Nation level, loss of language, loss of homelands, relocation, discrimination, and premature deaths impacted elders (see Figure 1).

### Childhood stories from elders

#### Living the traditional Navajo life—Phil Steven, Navajo Elder

“I started doing stuff at a young age. Probably 6 years old. We had livestock, you know, sheep, horses, goats, and I took part in that, living in a one-room hogan with a dirt floor, a hogan that is made out of mud. We all slept in there, and we share everything … taking part and taking care of the hogan and the livestock. It was a daily task each morning, early in the morning, being woken up by our grandparents and our parents. Waking us up to go run in the morning before the sun came up. After we ran, we came back and took out the white cornmeal. And then we went back out and we … after that we washed our hands and face; that was it. Fold up your bedding, and we never had our bedding inside the hogan; it was always taken out. Because we shake it out, they say you shake out the laziness, all the sleepy people, you shake them out of your bedding and start a new day fresh. That is what we were told in the morning. Then it was on to go get the horses. First thing, they were hobbled, bring the horses back. After we did that, I would take out the sheep. If it’s wintertime or snowing, you still took out the sheep. We live a harsh life. You know, we were out there, herding cows too. We would take them out; we could not lose eye on our cattle, let them roam. We took part in that kind of lifestyle every day. Every single day, it was the same thing, the same task, over and over. And that is how I grew up. I know I grew up in a harsh environment, where we had to do things … do the things, if there was something wrong, you were yelled at, spanked, hit with sticks or something … growing up, my parents and my great-grandmother were mean. Me growing up that way, I was blessed because it made me stronger. In my heart and in my mind. Even though I live in a modern society, I am so proud of my old ways.”

#### Tenant farming, early mornings, and education—Dolores Subia BigFoot, Caddo Elder

“Well … we were … laborers. So, we were tenant farmers, so my father … we lived in a house that the landowner had, the farmer had, and then we worked for him. So, he was our employer. And he grew cotton and peanuts. So, cotton had to be chopped, and then it grew, and then you had to pick it and bale it.

And peanuts, you had to irrigate it. Hoe it. Dig it. Sack it, and haul it. Dry it. You started early in the spring, with cultivating and getting the ground ready. And you planted, and you tried to keep on top of the weeds … there was a lot of Bermuda grass, so you were always fightin’ baby grass. And I remember getting up and being in the fields by about 5 o’clock in the morning, when it was daylight. We had to move pipe, and it took about two hours. So that was irrigating those, the, you know, crops, and then we would go home and change clothes, and we did not have indoor plumbing or anything, so we had a pump and well water, and heated up our, hot water on the stove. We were not really taking baths in the mornings or anything. So change clothes and then get back out into the field and … and work all day, … and then stop about 3 or so and move pipe from 4 to 6 again, and then go home and eat. It was a lot of hard work.

And so … I got my degree. I was at a master’s level. I was working as a mental health therapist with a residential ranch in Montana … this is where the kids lived on the ranch and went to a Tribal school. We offered services, but we also provided supervision for them. So, there was a couple that was the house parents, I was the mental health therapist, and there were assorted adult workers for supervision, and then a ranch foreman. And I remember … So, there I was, I considered myself a therapist…remembering all of those times of getting up before 5 o’clock in the morning to be out in the field at 5 to move pipes and thinking, this is not how I want to live my life.

And then, getting this job as a master’s level therapist, and then after I got the job and was working, they told me that I had to go out and work with the kids and move pipe with them, that I had to irrigate.

I was thinking to myself, this is why I got the degree, so that I do not go out and have to haul pipes. I remember very distinctly, telling myself as a … as a 12-year-old, that I did not want to live a life that was based on manual labor. I mean, it was my total motivation for going to school. Going to school was a release because when you were home, you were working all the time. In the wintertime, we did not work as hard, but, you know, spring, summer, fall was pretty … a lot of … lot of long hours. It kind of is amazing because there’s a period of time that we got paid $1 an hour for working. You know? And that’s … a dollar an hour, you know? Of course, gas was 35 … 25 cents, or 35 cents a gallon.”

#### Indian life, cow camps, and big wild cats- Gail Raines, Crow Elder

“So, we moved out to the cow camp, which, as the crow flies, is probably about 30 miles from here [Pryor, MT]. But it’s out, it’s way out in the foothills, in fact, they call it ‘The Little Mountain That Rises Right Behind It.’ My dad went to work for this big outfit, The Antler Ranch, it was all over the reservation. And he would ride off and be gone for like, if it was time to brand calves, he’d be gone for like six weeks. If it was shipping time, he’d be gone about that long, too. He′d ride, take his string of horses. So, I lived my life for like three years in a couple of different cow camps, and I had a string of stick horses. I had my mom buy the same color of stick horses as my dad’s horses. So, I had a red horse that matched his Sorrel, a yellow horse to match his Palomino, you know, like that … I’d ride my stick horse around, and I was just always outdoors. And I remember that, of course, we did not have electricity. The only way we were entertained in the evenings was that my dad told really great stories; he was a storyteller. And he always told me the romantic stories about Indian life. And then, because I never had a playmate, I mocked being a woman, a wife, a mother, somebody baking bread in the kitchen on my little table, or I’d change, and I’d be Jig, who you know, still that’s my nickname. Jig was the man counterpart, but I’d be out riding those stick horses or out riding a real horse, or, you know, doing all these things that I knew my dad was out there doing. Or I followed my mom around. That was how I learned. But that hooked me so hard … I lived out there, I mean, I was a little bitty thing, and I could stand out there, and that tall prairie grass just waving in the wind. I just remember that light, like I never forget that. If you turn and look that other way, then I’ll never forget that big mountain rising up. And just like living with the creek running right through there, I had no idea how perfect that was at the time. But it was. And, um, yeah, I made friends with horses. My cat, my big wild cat, black and white, it got to be as big as a bobcat, eventually. We moved from one place to another, many miles away- three, one, two- yeah three times he [big wild cat] followed us and found us; from Pryor Creek to Scott Creek; from Scott Creek to Garyowen; from Garyowen back to Pryor. He just ran wild, and then would come home and find us back where we were. And so, I’ve always thought a lot of that, um, intelligence of animals. It’s really meaningful now to realize when I was a kid how much I really did learn. Just from communing with them and not having anybody to play with.”

#### Wounded knee, AIM, goon, and survival—Ruthie Cedar Face, Lakota Elder

“I remember, I think I was about 7, maybe 7 or 8… there was … still a lot of AIM and Goon stuff going on. There was still a whole lot of FBI activity on the reservation because they were still looking for people who were involved in the occupation, and we ended up, you know, just seeing the aftermath of all of that. And in our little housing area, we had people who were claiming to be AIM and people who were claiming to be Goon, and they were drinking, and so it ended up becoming fights that happened. But what happened was, it wasn’t just fights, it was gunfire. And so, us kids learned how, when we heard a gunshot go off, we learned how to drop to the floor. And so, in a sense, we all had suffered, like, PTSD, I think. Because in the summertime, my mom had sheets on the line from washing, like she washed sheets and hung them out, and the wind snapped the sheets, and we hit the ground. It was just like an automatic response. In the middle of the night, when we were sleeping, but we heard gunshots, or what we thought were gunshots, we’d hit the floor. So it was, like an automatic response for us. I’m sure some of the people, some of the kids that are still alive and our age, they probably remember a lot of that stuff. But I remember one day, we were over at my friend’s house. She lived two houses down from us, so me and my sisters were down over there playing with her, because she had everything. She had the toys and the bikes and everything, so we were over there with her, and her mom came in, and she said, ‘You, you girls, you better go home. Go home now, get home now.’ So, we headed out, and there was a playground below her house, so we went down there and was playing on the playground. And pretty soon, we heard these cars screeching, and that woman … and my friend’s mom was hollering, ‘Go home! Now! Go home!’ And then my mom was by the house, hollering for us to get home. Come on, girls, now, now! So, we ran, and my sisters were running in front of me, and we ran. And I think it was probably almost like a, maybe about 100 yards from the playground to my mom’s. Probably like a football field long. And there was a trail. So, we ran that, and then we had to run up the hill to the house. And so my sisters ran that, because I was always the chubby kid, and so, you know, I didn’t run as fast as them. And as I was running up that hill. I tripped, and I fell, but at the same time, somebody shot a gun, and that bullet hit right beside me. And my mom screamed. And I could hear her scream, I could hear her call my name, and I was like, ‘Oh my god, what’s going on?’ And so, I put, like, I peeked up at her, and she was running towards me, and she grabbed me and ran with me back to the house, and she thought I got shot. She thought I got shot. So, these cars were cruising around, shooting at each other, claiming to be AIM and Goon, and they were just a bunch of drunk assholes, basically, you know, using that as an excuse. And so, things like that, incidents like that happen throughout the post-Wounded Knee years. And so, we end … you know, we ended up, you know, dealing with that, and so I never could call myself an AIM supporter, or I never could say I was a Goon. I was, I just never identified with either one of them, because in my eyes, a lot of them weren’t very good people, you know. And so, like, now I believe, you know, the things I believe about, yeah, during that time, identity was brought back. But then there were also a lot of other things that were brought back. You know, they taught our young people to be proud of who they were as young Indigenous people, but at the same time, they taught a lot of the younger ones to be fearful of a lot of different things.”

#### Living off the land, feeding the community, farming, and loss of language—Bill Snell, Crow & Assiniboine Elder

“Well, you know, for me, I guess I kind of grew up kind of like a, uh, I’m a ‘Sawyer’ type. Pretty much grew up with nature and my dogs, and I did not have a lot of time to socialize with friends and other things because I grew up on a farm and ranch on the Milk River Valley and Fort Belknap. And I think the thing that kind of helped define me as a young person was that I really looked at nature as my teacher. And I think I grew up, you know, hunting and trapping and providing food for the valley. Because back then, it’s something I really loved to do, and I do not know how it got into me. Maybe it’s because of a spiritual commitment. I do not know. But it was really important for me too, as I was able to hunt and fish and trap and those kinds of things, but also to help all the individuals that lived in the valley. And so it was … it was quite an experience to help feed those elders and others that lived in the valley who were Native and sustained themselves. And so, I just helped provide that, and I went around, went through the valley, making sure that different families had meat or even vegetables because we grew big gardens; and you know, give them potatoes and give them beets and squash and other things like that. And being able to take it to them and then see the smile on their face when they received it, and how thankful they were. That was really rewarding to me, and it kind of defined me as a child as to what I really like to do. It was just neat to see that. It’s not just nature and those kinds of things, but what resulted from my ability to work with nature to help others as well. I grew up in a boat and a canoe and ice and wilderness. It was pretty much myself. And taking care of the farm and ranch and the things. I definitely learned work ethic, that’s for sure. And my mom and dad had to supplement the farm and ranch, and so they worked as well. I would be out trapping when it’s 20 below and doing that kind of thing and hunting and other things. And my parents never did check on me. I mean, they truly trusted me to be safe. They would ring this bell or whistle–we kind of communicated by whistling–and so I knew supper was kind of ready if I was close enough to hear. But it was always me and my dogs and that was it. And so that’s how I grew up. It was interesting because when we started farming and ranching, and we were transitioning from horses to tractors, and so you know that whole 1950s era to ‘60s, we could not afford a major big tractor. We had a small tractor called a ‘John Deere B, ‘they called it. And so, they used the horse-drawn equipment and hooked it up to the tractor. So, they would cut the tongue off, shorten it, hook it up to a tractor, and then we would pull it with the tractor instead of horses during that time, which was pretty interesting. So, pulling things that actually are horse-drawn versus tractor-drawn created some problems. But, you know, you do with what you got. I was born in 1951, so December, and so I think probably around 1959 is when I started driving. I could barely reach, you know. We had these old trucks that had–they called them throttles. Right, so you would start them up, and then you could not reach the pedal when you are too small, and so you’d have this throttle. You put it in gear and have this throttle, and you’d pull it so you’d go faster or slower, and so you’d, you know, you would feed the cows that way, and throw the bales off, etcetera.

My mom and dad, they all spoke Indian. And there’s times on the ranch I would come around a corner and they’d all be speaking Indian. And then when we [the kids] would come around, they would just stop. And it was like, you know, it was like, why did you stop? And I’ve always wondered that. And as I’ve assessed it and analyzed it a bit as I grew older, I realized that they were trying to protect us. Because at that time you are kind of beat and spanked and stuff for speaking your language in boarding schools. And they did not want us to go through that, so I think that’s the reason they did–when we would come around, they would just stop talking and then just start trying to speak in English or sign language, and I think that’s what happened.”

#### Reservation life, kinship, discrimination, and life-changing events—Clayton Small, Northern Cheyenne Elder

“We had a home, we had horses, we had cattle, and that was all my playground. I had thousands of acres to, to play out in nature. And I really needed that because of all the trauma that affected my family when I was a child. And so, I could saddle up my horse, and I’d be gone all day, riding with my cousins. Getting in trouble, I had several broken bones, legs, wrists, because we were unsupervised, and we were just out having fun every day on the res. As they say, getting Rezy, you know? And my parents had to go look for me, you know, at 9, 10 o’clock at night. That’s time to come home. And so that was really a blessing. It was, it was my way of protecting myself from our family dysfunction when it comes to violence and substance abuse, and living in poverty. I really did not think of it that way as a child, but I was thinking that everybody lives like this. Everybody around me, you know, has this lifestyle. And that was pretty much true if you lived on the reservation. But you travel off the Rez, and life is different. And I did not see that difference until I was probably in high school. So, for me, I would listen to our neighbors, my mother’s uncle, August Spotted Elk, who lived across the way, and his sons were my first cousins, and we just, you know, we were besties. And we were either playing on the dirt basketball court or riding our horses. And at night, I would go to sleep to the Spotted Elks, tying up their water drum and singing Peyote songs into the night. And so that was a protective factor for me. Having access to my horses, to nature, to my cousins. Kinship. Kinship is important, you know, extended family. And when you live on the reservation, you have that. So, I had horse medicine … I was getting therapy without even knowing it. You know, being close to nature and being close to horses. And my family, my father comes from ranchers, from hard-working people. That was another thing I learned living on a ranch is that you have to work. You have to work hard, and you have to appreciate what you have, you have to be respectful, and you have to honor your family in the best way that you can by setting a good example, because you have a lot of relatives, and you know, they are watching you.

Oftentimes on weekends, our family, our mom and dad would go out, and they would drink, and they’d get wild, and they’d come home late into the night, or not come home. And I remember as a child, lying in bed thinking, are they gonna come home? Are they gonna get arrested? Are they gonna get into a fight? Am I gonna be an orphan? And you know, I had deep thoughts about that regularly. And so, I had to compartmentalize, just kind of put that kind of trauma to the side, because I did not have the skills to deal with it. So, that kind of fear of abandonment and rejection and survival.

I’m a light-skinned Cheyenne. And with that, I realized much later that it was a privilege. I did not suffer as much racism as my darker-skinned relatives and friends, but I saw it. I saw it constantly. I attended a high school in a border town off the reservation. Half of the kids were white, half were Native. And I got to see that, you know, and then shopping off the reservation, border towns, you know, racism just … it does not feel good, whether you experience it or you witness it. I realized that because I was a light-skinned Native, I did not suffer as much as my friends. One of the stories I heard much later about my older brother, Clifford, who’s deceased now, was that he dropped out of high school. He attended a Ray Indian School and a boarding school. And I found out much later that one of the reasons he dropped out was because of the sexual assault that was occurring to young men over there in the boarding school by … by the priests. So that was one of the traumatic incidents that he had to carry with him. Yeah, you know, I am a work in progress, and at some point, you turn from being a survivor to being a warrior who can say to yourself that no matter what comes into my path, I’ll be okay. We moved from our ranch, from our homestead, when I was a freshman in high school. And part of getting that new home in Lame Deer was that we had to work. We had to spend so many hours on the construction of our home. That was, like, our down payment. So, as a young teenager, I was pounding nails, putting roofs on, laying tile, and painting so that we could afford our down payment for our new house. And so, moving from our allotment to cluster housing in Lame Deer was a huge change in direction for our family and for my life. I missed being out there on the ranch. I missed spending time with horses, rodeo, and nature, and so that was restricted. You know, my life shifted. But I became, you know, I was blessed with being an athlete. The Creator gave me that gift, and I worked at it because I saw that for me to be successful, I needed to graduate from high school, needed to go to college, and that was a way out of poverty for me, and sports, getting a sports scholarship, was another thing that I established as a goal. And so, I worked hard at that. And you know what? It kept me out of trouble, except that I still hung around with knuckleheads, and I could be one too. I remember that by the time I was 19 years old, I was a survivor of two car wrecks. In one of those car wrecks, two of my friends were killed. That was my wake-up call. It was like, you know, if you want to live to be a man, and a father, and a grandfather, you have to make some better decisions. By the time I was 19, that clicked for me. That you need to stop hanging around with knuckleheads, you need to make better decisions, you need to focus on your education, focus on your sports, and get on with life. You know, that was a major crossroads for me, because in reflecting back to my class of 30 students that I started off at Lame Deer Elementary with, there are maybe five or six of us that are living today. And their deaths were tragic, and they were unnecessary. I’m thankful that at age 19, I took a different direction in my life, my behavior, my attitude. I’m not a cat with nine lives, you know? If I’m not careful, I’m not gonna be around very long.”

### The wisdom you want to pass on

This section presents wisdom stories from the elders interviewed. All elders, at one point in their interviews, talked about the importance of story, telling it, sharing cultural and traditional ways, and keeping the Indian way of life intact for future generations. For some elders, this was the first time they had considered what wisdom they wanted to pass on to the coming generations.

#### Stepping back to see wisdom in story—Dolores Subia BigFoot, Caddo Elder

“Yeah, it’s been a conversation that’s been … going on … in various ways, and in my head. I think we always want to have conversations about what’s important to us. But sometimes we don’t have the wisdom to know what’s really important to us. And I think the other part of that is just assuming that stories about the past are just stories about the past. And, that they’re sort of being handed down. Sort of to preserve the stories, but not necessarily as life… learning, or problem solving, or whatever. Then you start to recognize the bigger picture that surrounded some of the things that occurred. So, for example, just talking about losing our … Native language. And initially it was, well … It was because, you know, mom was a fluent speaker; the boarding school distorted her whole sense of self … our parents didn’t teach us, and they wanted us to learn English, they thought that it would make it easier for the next generation. But what not having the language did is it distorted our sense of connection to the culture and who we are. And then you step back a little bit, and you realize that her earlier experiences tainted how she raised her children. And then you step back a little bit more, and then you realize that, oh, it was the boarding school era, to the eradication of cultural knowledge. And then you step back a little bit more, and you realize about colonization, and the historical trauma, and then you step back a little bit more, and you realize how the, you know, a bigger system has impacted, not only our small Tribal community but other communities and shapes the world in various ways. This is when we start looking at structural racism and contortions… especially what we see in the current administration. So, as we think about these little individual family stories, we don’t always understand how those links are there. But then, when we start understanding or appreciating more those small links, then we start to say, you know, that’s quite remarkable. That’s a lot of tenacity, or there’s a determination on their parts, and then you start taking pride in small efforts that you didn’t realize how harsh those circumstances were at that moment, and then you get to pass on that history.”

#### Keep praying—Dolores Subia BigFoot, Caddo Elder

“Well, the wisdom is to keep … to keep praying for the next generation, and to, and in those prayers, tell … of who, in the past, prayed for the future. And how they prayed, and how they acted, what they fought for, what they were concerned about. What they saw was important, and how they navigated life even when it was very limited.”

#### You have a voice—anonymous Elder

“I think the wisdom I want to share is that we have a voice. And we must honor that gift, whether it’s, you know, academically trained, or it’s, you know, experiential from, you know, 70 or 80 or 90 years of living in our Tribal lands, in territories, in pueblos. We need to tell our history. One of the things we do kind of grieve for is that our history has never been told correctly. How do we tell it correctly? You know, how do we right the injustices that have been done? Historically, we have an eye toward the future for the next generations. We have the instructions. We know what we’re supposed to be doing. We have to interpret those instructions for where we are right now in history. I also think that some of the things that I struggle with, even as a grandmother, are, how do we maintain whatever level of cultural awareness and practice that we have in our lives? How do we maintain those and interpret them as engaging our youth? You know, why should they be paying attention to what happens if we lose our language? Who then are we, as the people we are? The people that our creation story told us we are. Also, I think that we have to look through the lens of our children. And figure out what it is that they need most? They need us to let them know we love them, that we are proud of them, that we share a common history with them. That might be the thing that breaks that barrier between the old and the young. I think it is the shared history. Being proud of who we are and acting on that pride. Not just taking pride in someone else, but actively contributing to the pride in who we are as a particular people. My feeling is that if we can keep our children and provide them with a strong cultural foundation, over time, they’re able to listen to us, even before they’re born. Things that you say are understood and absorbed into that growing fetus. If we can ensure that our children are being given the foundations of our core cultural value systems, our belief systems, and our stories. And even if, along the way, they stray away from that, and they are involved in things that could take them away from us. If we can keep them alive long enough, they will come back to those core cultural values of their youth. They will. I firmly believe that. The thing is, how do we keep them alive long enough for them to come back to that?”

#### Go back to the old ways, share your stories about how to live, walk in beauty—Phil Steven, Navajo Elder

“Trying to get back to our old ways of living. Share that story. Share it with whoever is close to you, whoever is going to keep it going. Share it with them. Tell them how we lived, share that story with them, and what they mean to us by waking up early before the sun. Waking up before the sun, you will not age fast, they always said that. During the winter, go take a snow bath, you know. Wash your face with snow and go out there barefoot. Let your feet touch the snow. Wash yourself down, and that is how we grew up. Every day during the winter, going out in extreme cold. Just our cutoffs and no shirts. We ran like that in the morning in the cold, so that you could be strong. You could not age. You could stay healthy. We were told that was the story, that was the story they told us to do, so we done it. During the summer, we run on the hot sand barefoot. Barefoot. Right at noon, they make us run. And nowadays, we do not do that, and that’s the reason why we are so obese. You know we suffer a lot. That is the one thing that I think, where we have to … nowadays, like with me. I am not living on my reservation. Because our prayers say we are supposed to stay within the four sacred mountains. You can go outside of that. I still respect the rivers and streams, like I am still living on the Navajo reservation. Wherever you go, you have to respect the land. Some people might say you are not living a good life. Whatever you do, no matter where you are at, just do what you have to do to stay on that white corn meal, that road of life, that yellow and white, the corn pollen. The beauty way. Walk in beauty; you can do that anywhere in the world.

The one thing about marrying other Tribes is that you have to accept their cultural way, and you have to accept theirs. Both couples have to accept their beliefs, they both have to practice, it’s not just one way, and they both have to respect, then you will be living in the beauty. Nowadays, people disagree on things. You hear on social media. That is the Navajo way. I always disagree. You are over there, you are doing that. I disagree. It is up to the person. They are living a good life. They have to continue using whatever they were taught to use. We were taught to use the yellow cornmeal, the white cornmeal; those are the things we carry on with us to protect us.”

#### Love your family, limit technology, protect the land, and medicines—Gail Raines, Crow Elder

“The wisdom is that Natives, Tribal people, have always been and always will be marginalized by a greater society that will never agree to making reparations as completely and generously as did our forefathers in agreeing to false promises and broken treaties. The more we rely on others to make things right, the further we get from our identities. In the way of the world, I would say, fight to the bitter end for freedom to practice your own natural way of being. Seek the Maker’s guidance every day. Keep your family close. Let them know you love them, that you believe in them. Get this schooling figured out or go back to the old ways. We need to be more involved in our children and grandchildren’s upbringing to keep hope and achievement going. To keep the beauty of the culture alive and be, you know, open to working together to strengthen community and learning how to work on the things we do not talk about. I wished- here’s the thing–I’m wise enough to know that people need to unplug. They’ve lost the feeling for the land, these people now. I used to laugh when my mom would say, “You’ve got to show people; the school has to bring kids out so they can see family farms and ranches, so they can see what goes on, where the animals are, where their food comes from.” And I used to think that was so funny, that there might be anybody in the world that did not know that their eggs came from chickens, or milk came from cows, but now here we are. Here we are. Not only that, but they are fighting us for killing animals, or even anything, anymore, beyond reasonable. We must keep our rights to hunt and eat wildlife, including Bison. There is a great push to make the western part of America home again to the bison, a healthier choice for Native Americans and for the environment, however there will be a political war in taking back the Bison territory from the cattle industry that instigated the annihilation of the bison to begin with. It would be wise to be ready to capitalize on whichever industry wins the West, in my opinion. I have given more thought to what we call noxious weeds because so many broadleaf plants have medicinal qualities that Natives and Asians have always used, and pharmaceutical companies keep a secret from us. I live in a different world than I ever thought I would be, for sure. I guess I wish I was more wise.”

#### Deal with pain, practice ceremony and self-care, live in balance—Ruthie Cedar Face, Lakota Elder

“I just feel like, for anybody coming up, you know, take care of yourself. Not just physically, but mentally, emotionally, spiritually, whatever way that looks to you, to take care of yourself, because as you get older, those things can help you. If you have somewhat of a balance in those areas, you’ll be okay. Because I do not believe there’s total balance. I mean, that’s perfection, and there’s no perfection, you know? So, take care of yourself, because a lot of times, we do not deal with things we need to. We do not address areas that we need to. And it sits, and it becomes something else. Whether that’s a physical ailment, a mental ailment, emotional, or even spiritual. So that, for me, that’s the biggest thing that I can say to anybody younger, as you grow, take care of yourself. Because I know in my own … in my own journey, I did not know how to do that. I did not, and I thought somebody else’s ways of doing things would work for me, but it did not. I had to find those things that helped me take care of myself. So physically, you know, I deal with sicknesses. Because of my not knowing how to handle stress, or not wanting to deal with stress, or not dealing with things, I feel like it became a physical ailment for me that I had to learn how to manage today, that I had to learn how to take care of myself. I’ve seen friends not deal with things that they had to deal with that were emotional or mental. And it became depression, it became things that were not, you know, that they have to battle on an everyday basis, so yeah. Self-care is probably the biggest thing that you do for yourself. Especially if you are, you know, taking care of other people.

I think it’s really important to get stories out there that need to be told, you know. It’s honoring my story. Where I’m at today is because of a lot of that stuff that I went through as a young woman, as a child, you know, that type of thing. So, it really did help shape me. But along the way, I had to learn how to take care of myself so that I wasn’t a statistic. And so, I turned 58 this year, this month, and you know, I’m thankful, because a lot of my friends and relatives did not make it past 40, yeah. And so, I’m not a statistic, and I’m thankful for that. And it allows me to do the work that we are doing. Like, we do sexual assault healing camps for women, that are culturally based; and we have done one last month, we have done one for teen girls, this spring. There are healing camps. I’m helping to develop and plan, an elders healing camp this coming year, and then I help with the children’s healing camp. I do, like, the womanhood ceremony, the rites of passage, the Išnáti, we are gonna do Throwing of the Ball. I was able to take part in a ceremony called the Paints Her Red Ceremony, and that’s a ceremony for elder women when they go through their change of life, and then they move into that quadrant of their life, that fourth stage. I was honored and able to go through that, and then I was honored to be able to help 3 more groups go through that ceremony. So, you know, it’s like a really … I’m in a really good place in my life now, probably better than it was when I was a young woman.”

#### Pray, help people, commit to something—Bill Snell, Crow & Assiniboine Elder

“Oh gosh, you know, there’s so much to say about wisdom. It’s something that I ask for every day, and I pray for wisdom. And it’s important to me that I do that. One of the greatest things that’s helped me is to accept all people the way they are. I think that you never discriminate, you never judge, and all people have certain needs and need to be loved. Committing to that is important because it never fails, it’s always there. And it becomes part of your way. But the bottom line is really about respect, honor, and acceptance. So important. And also, you know, trying to teach my sons and us, make sure we have a purpose, because without a purpose, you know, what do you do? We’ve got to commit ourselves to something, and it’s important that we commit ourselves to helping people in general, whoever it might be, in any atmosphere, in any kind of work we do. It kind of gets ingrained in you, I guess, when you’re young. It really does come with your parents’ teaching. But I think the other part of our life that we cannot ignore at all is our spiritual life. You know, when I think about it we as American Indians still pray every time we have meetings. And I think it’s not just a prayer, I think it’s so important that it’s not thinking just about spiritual things, but we actually live them and put them to work for us and ourselves, and I think that’s critical. So, praying is one thing, doing is another, and it has to go together, you know. Faith without works is dead.”

#### Practice ceremony, forgive, heal from losses, be generous, help others, seek happiness—Clayton Small, Northern Cheyenne Elder

“When I realized that I needed to work on myself, you know, whatever career path I had, I had to put on hold. I went home, and I started our ceremonies. I went fasting at Bear Butte for 4 years. Sundanced at our ceremonies in the summer, got more involved with our sweat lodges, our peyote meetings, and that was a major shift for me to embrace our culture, to embrace our spirituality and our ceremonies, and it helped me to see where I needed to go with my life. It wasn’t living as a white man in mainstream America, you know? It was embracing our culture and serving our people. I had to work on my self-care. You know, and as I started, living towards midlife, getting to be 40-ish, it was forgiveness, forgiving my mom and dad, forgiving myself. Strengthening my spirituality, embracing my culture and cultural resilience, you know, using our Native culture to overcome trauma, tragedy, and threats. Knowing that that was probably my greatest source of strength, and to believe in it. You have to believe, you know? So, here’s a thought from my friend Bim. And those who were seen dancing … they were thought to be insane by those who could not hear the music. So I was able to hear the music through our culture and our beautiful, Cheyenne way of life, which sustains me today. I learned how to take care of myself and the importance of self-care. I worked hard on being generous, on being of service, and helping our people, and to have healthy relationships, that in a lifetime, you may only have a handful of friends that you can call good friends, and that you needed to work at it, foster that, and develop that. I learned how to spend some time on the losses in my life. To take time to grieve. And to pray, and not to put those losses on hold for too long. So, in 2013, I lost my mother.

She was 86. In 2015, I lost my dad, he was 91. Three years ago, I lost my son. And so those were huge losses. You know, becoming an orphan is a tough challenge for a child, no matter how old you are.

And then losing your own child was overwhelming and changed the course of my life forever. The reality is, these are bumps in the highway that the Creator puts in our path, and we have to use our strengths to navigate and to work through them. And I guess I’ve learned as an adult and an elder, that we need to, in spite of all the challenges, we have to seek happiness. We have to look for it, and we have to work at it. We have to be generous, and we have to reach back and help those who are struggling. If we own the story, if we own our story, then we can write the ending. Right?”

## Discussion

In this study, we present stories from Indigenous elders that reflect on their childhood experiences and the wisdom they want to pass on to future generations. These narratives demonstrate resiliency and strength across the individual, family/community, and Tribal/Nation levels. Most elders described deep connections to the land, nature, animals, culture, traditions, language, and reservation life. Their stories emphasize the central role of family and community in shaping both their individual and cultural identities, as well as in fostering knowledge of healthy relationships, kinship, and traditional ways. Collectively, these narratives underscore the Indigenous life course and traditional values and teachings, such as cultural pride, spirituality, service, resilience, freedom, and self-sufficiency. Elders’ childhood stories reveal a profound connection to the land and a reverence for culture and kinship. Elder wisdom stories give readers insight into how to live in the present day, and the legacy elders want to leave for future generations.

This study was designed to support the transmission and continuation of Indigenous knowledge systems, lifeways, practices, traditions, values, and experiences that are often lost or never recorded. This study illuminates the life stories of elders who possess deep resilience and strength and, at the same time, experienced the devastating impacts of colonialism and the loss of land, language, values, and family systems. Elders provide insight into their lives and what it was like to be an American Indian living in the US during the 1950s–1970s. All elders mentioned the importance of oral traditions, stories, and histories in preserving the indigeneity of AIANs, America’s First Peoples.

Elder Clayton Small reflected on the sexual abuse that stemmed from these colonial institutions. Elder Ruthie Cedar Face talked about what it was like to grow up during the Wounded Knee Occupation and to live with AIM activists. The AIM movement was another pivotal moment in history, with reverberations in today’s social justice movement, Tribal governance, relationships with federal law enforcement, and distrust of non-Indian institutions. Elder Phil Stevens talked about living the Navajo traditional way of life in a one-room hogan with discipline and routine. Elder Dolores BigFoot reflected on life as a tenant farmer, the hard work and physical labor she endured, while setting her sights on earning a master’s degree and a doctorate to escape poverty. Elder Gail Whiteman remembered life in cow camps, riding thousands of miles horseback on the Crow reservation. She lives on these ancestral homelands and urges people to disconnect from technology and protect the traditional medicines. Elder Bill Snell’s early childhood on the Fort Belknap reservation, hunting, living off the land, and freedom give insight into reservation life nearly 70 years ago.

Elders’ deep wisdom and legacy are reflected in what they wanted to share with readers, a call to action about how to live and what they will leave.

*Keep Praying - Dolores Subia BigFoot, Caddo Elder.*

*You Have A Voice—Anonymous Elder.*

*Go Back to the Old Ways, Share Your Stories About How to Live, Walk in Beauty - Phil Steven, Navajo Elder.*

*Love Your Family, Limit Technology, Protect the Land, and Medicines - Gail Raines, Crow Elder.*

*Deal with Pain, Practice Ceremony and Self-care, Live in Balance - Ruthie Cedar Face, Lakota Elder.*

*Pray, Help People, Commit to Something - Bill Snell, Crow/Assiniboine Elder.*

*Practice Ceremony, Forgive, Heal from Losses, Be Generous, Help Others, Seek Happiness - Clayton Small, Northern Cheyenne Elder.*

## Limitations and strengths

There are many strengths to the elder-centered research methodology presented in this study. However, some limitations in use, interpretation, and application should be noted. First, due to the manuscript’s length constraints, the stories are not included in their entirety. These stories cannot and should not be generalized to other Indigenous populations, communities, cultures, and traditions. Second, elders could share any story they wanted to about their childhood; this was not limited to a specific topic, time, or location. Therefore, stories of childhood cover ancestral wisdom, conflicts and trauma, land, and ways of being. Stories are not complete stories or accounts of a particular time and event, and readers should not make assumptions about what happened before, after, during, or because of these events. Readers wanting more should seek Indigenous history resources written by Indigenous people. Third, language limits the meaning, memory, feeling, and heart knowledge that comes from these elder histories and wisdom. While this study aims to centralize Indigenous wisdom, decolonize research approaches, guide readers toward ways of living, and transmit intergenerational wisdom, we recognize that Western academic publications and research limit how information is accessed, interpreted, and used. The use of Photovoice, videos, audio, art, and other visual methods is necessary to extend the reach of this work and to honor the knowledge systems and oral traditions of Indigenous people in the future. Fourth, the first author’s position as a researcher and existing relationship with elders undoubtedly influenced the stories elders shared, but in a deep, meaningful way. Finally, some may view internal consistency as an issue, given the nuances involved in explicating the analytical processes used to present the stories and validate the visual model. In this study, replicability and internal consistency are not the primary goals of elder-centered research methodologies in Indigenous contexts; however, the elders reviewed, edited, and validated the visual model after reviewing all stories in their entirety. Elders are the knowledge keepers who put forth the final model. By honoring Indigenous worldviews, epistemologies, Indigenous data governance, and the CARE principles, we believe this visual model is imperative for conveying the wisdom of these stories.

These stories, authenticity, and vulnerability were possible because of the relationship, respect, trust, and enduring connections with the first author. Unlike Western research, where the funding agency, principal investigator, or university owns the research and data and controls what stories are told, this research, these stories, and the subsequent wisdom offered are a gift of generosity to all readers. Here, Indigenous voices, wisdom, and love trump everything, as one Lakota teaching reminds us: “We are all our grandfathers’ grandchildren.”

## Conclusion

Elder stories elevate the power of lived experience in public health as a foundational component of existence as spiritual beings on this great Turtle Island. Public health may draw on lived experience as a teacher and a deep source of knowledge when developing programs, policies, and interventions. This is already underway in Indigenous public health spaces, where individuals with lived experience provide support and guidance to others with similar experiences (33). The implications of these stories and this paper are far-reaching; they remind readers that we are the sum of our ancestors, our histories, and our teachings.

Public health programming, policies, and interventions are often driven by quantitative measures that identify deficits rather than strengths. Public health could benefit from decolonizing these deficit-based methods to be more person- and elder-centered and grounded in the lived experiences and values of Indigenous elders presented in this paper. Public health policy could also benefit by relying more on lived experiences and the wisdom of elder teachers. Policies created by the US government over time have been ineffective in promoting the collective well-being of Indigenous people. They continue to have the lowest life expectancy of any minority group in the nation (2) and experience the lowest level of public health funding and support of any group in the nation (34).

What history tells us and will continue to show us is that we must step back and be intentional about how we collect data, who we involve in data collection, who and what is driving public health policies, and the wisdom that is used to create them. We must also consider who has the power to make decisions for Indigenous people (35). Here, Indigenous data sovereignty, Tribal self-governance, and health sovereignty are key considerations when planning and implementing health programs, education, and community-based Advocacy research (36) and interventions.

What stories do you have to tell? What stories have you collected in your memory that inform how you live and see the world? What stories have your elders and ancestors shared with you that you rely on to live a balanced and healthy life? These are the fundamental questions we must ask ourselves as we walk in beauty toward the coming generations. Passing down wisdom through stories is integral for public health and well-being for all. It is now time to pass on the wisdom of the elders.

## Data Availability

The datasets presented in this article are not readily available because tribal elders requested stories/raw data not be available. Requests to access the datasets should be directed to kelleyallyson@gmail.com.
